# Improving performance of the STochastic Engine for Pathway Simulation (STEPS)

**DOI:** 10.1186/1471-2202-12-S1-P58

**Published:** 2011-07-18

**Authors:** Weiliang Chen, Iain Hepburn, Erik De Schutter

**Affiliations:** 1 Computational Neuroscience Unit, Okinawa Institute of Science and Technology, Okinawa 904-0411, Japan; 2 Theoretical Neurobiology, University of Antwerp, B-2610 Antwerp, Belgium

## 

STEPS (http://steps.sourceforge.net) is a GNU-licensed simulation platform that uses an extension of Gillespie's SSA [1] to deal with reactions and diffusion of molecules in 3D reconstructions of neuronal morphology and tissue [2]. In STEPS, the diffusion of molecules is simulated as diffusive fluxes between tetrahedral elements in the mesh, represented by a series of first-order reactions.

STEPS has been used in various research projects where its overall performance was considered adequate. However, as more complex models are being developed and investigated, total model simulation times became an issue leading to requests for a faster reaction-diffusion simulator. Here we discuss a number of strategies we have followed to improve STEPS performance at different levels.

The previous implementation of STEPS adapted Gibson and Bruck’s enhancement of the Direct method [3] with a *k*-ary tree structure. The overall complexity of searching and updating of this method is O(log*_k_N*), giving *N* as the total number of kinetic processes in the system and k as the branch width of the tree. Although this is a great improvement comparing to the O(*N*) complexity of Gillespie’s original method, the logarithmic dependency of *N* becomes a critical limitation of performance, particularly in simulations with high amount of tetrahedral elements and consequently large *N*. Recently a constant-time version of the Direct method with a slightly more complex data structure has been introduced [4], providing an attractive searching and updating complexity of O(1), independent of *N*. This method is implemented in the new version of STEPS. In the poster, I will present the validation of the new simulator by comparing the stochastic simulation results with analytical solutions. I will also present the improvement made by adapting the new method.

Several approaches to parallelizing STEPS have been investigated. In a realistic simulation, the state of the system, including molecule distribution, reaction/diffusion rates, may be frequently read and/or modified during the simulation. Some of these operations could be greatly parallelized in shared memory architecture, efficiently reducing the cost of data accessing. Previous studies [5] also suggested that the Direct SSA could be parallelized as a Parallel Discrete-Event Simulation (PDES). However, the performance of such a parallelization significantly depends on the model itself as well as the approach of simulation decomposition. Some examples of above methods will be present and discussed in the poster.
